# RBP7 functions as a tumor suppressor in HR + breast cancer by inhibiting the AKT/SREBP1 pathway and reducing fatty acid

**DOI:** 10.1186/s12935-024-03299-0

**Published:** 2024-03-29

**Authors:** Yue Yu, Zhihua Xu, Hao Zhou, Ruyan Xu, Jia Xu, Wenjun Liu, Yuxin Wu, Yue Qiu, Guangbo Zhang, Xue Huang, Yan Chen

**Affiliations:** 1 Department of General Surgery, The First Affiliated Hospital of Soochow University, NO. 899, Pinghai Road, Suzhou, 215006 China; 2 Jiangsu Institute of Clinical Immunology, The First Affiliated Hospital of Soochow University, Suzhou, China

**Keywords:** Breast cancer, RBP7, Prognosis, Fatty acid metabolism, Nomogram

## Abstract

**Background:**

Increasing evidence proves that RBP7 plays a significant role in breast cancer (BC). The present study was aimed to investigate the mechanism of RBP7.

**Methods:**

Western Blotting and qRT-PCR were performed for evaluating the expression levels. CCK8, colony forming, xenograft mouse model, wound healing and transwell assays were conducted to examine cell ability of proliferation, invasion and migration. Nile red staining and Oil red O staining were used for testing the lipid.

**Results:**

RBP7 was related to overall survival (OS) in patients with HR + BC. RBP7 protein was significantly decreased in HR + BC tissues and cells. RBP7 suppressed HR + BC cell proliferation in vitro and in vivo, and inhibited migration and invasion. RBP7 reduced fatty acid in HR + BC cells by inhibiting the AKT/SREBP1 pathway.

**Conclusions:**

RBP7 may function as a tumor suppressor in HR + BC by inhibiting the AKT/SREBP1 pathway and reducing fatty acid.

## Introduction

Breast cancer (BC) is the most common cancer in women worldwide and the leading cause of cancer deaths among women aged 20 to 59 [1]. Breast cancer cells with estrogen or progesterone receptors are hormone receptor-positive (HR+), accounting for 70% of BC [2]. For HR + patients, clinical guidelines recommend first-line endocrine therapy with or without targeted therapy [3]. A real-world study demonstrated that chemotherapy remains the mainstream first-line treatment option for HR + patients [4]. Recurrence caused by endocrine drug resistance and new adjuvant therapy are challenges in the treatment of HR + BC because 25-30% of patients with HR + BC encounter primary or secondary drug resistance [5]. On this basis, novel treatment strategies must be developed for HR + BC patients.

Cellular binding proteins (BPs) sequester and chaperone retinoids and other lipids to protect them from the cellular milieu, directing them to dedicated enzymes and receptors [6]. Cellular binding proteins influence the occurrence and progression of tumors by affecting retinoids in the process of uptake, metabolism, and function. Four cellular retinol binding proteins (CRBP) among BP have been identified to bind specifically retinol and retinal [7]. Epigenetic disruption of CRBP is common in human cancer [8]. Previous studies have revealed that abnormal expression of CRBP accounts for 24% of BC patients [9]. CRBP silencing by hypermethylation might be associated with some oncogenic signatures in human breast cancer [10]. Recent research showed that CRBP could suppress the PI3K/AKT pathway and the anchorage-independent growth of breast cancer cells, thereby inhibiting the development of breast cancer [11].

Cellular retinol binding protein 7 (RBP7, CRBPIV) is a member of the CRBP family [7], located on human chromosome 1p36.22, encoding a protein containing 134 amino acids [12]. RBP7 plays important roles in hypertension [13], adipogenesis [14], cold exposure and nutritional treatment [15]. Compared with other CRBPs, the RBP7-encoded protein binds all-trans-retinol with a lower binding affinity [12]. RBP7 is a PPARγ (peroxisome proliferator-activated receptor γ) target gene [16] and forms a positive feedback loop with PPARγ in hypertensive diseases [13]. A study from Elmasry et al. indicated that RBP7 was a strong prognostic biomarker for the malignant phenotype in colon cancer [17]. Tang et al. demonstrated that RBP7 and other immune system-related genes (IRGs) may affect the immune process of bladder cancer, suggesting a potential immunotherapy option for bladder cancer [18]. Reduced expression of RBP7 might lead to resistance to tamoxifen in luminal A breast cancer [19]. A study based on bioinformatics analysis predicted that RBP7 silencing by methylation promoted breast cancer through the PPAR and PI3K/AKT pathways [20]. However, the potential role of RBP7 in breast cancer has not yet been verified, and the mechanism remains unknown. Further research is needed to determine the role of RBP7 in breast cancer biology and its potential as a therapeutic target.

As an immune-related gene, the multifarious role of RBP7 in tumor is promising. Scientists have now identified many factors that play important roles in tumor immunity and tumor drug resistance. One study showed that constitutively activated ERK sensitizes breast cancer cells to doxorubicin by participating in the p53-EGFR-ERK pathway [21]. Singh S reported that through up regulation of p53 upregulated modulator of apoptosis (PUMA), TNFα induces p53 independent cell death [22]. Resistin was found to bind to TLR4 on the membrane of colon cancer cells and initiate TLR4-MyD88 dependent ERK activation, leading to cell arrest in the G1 phase [23]. Other popular factors such as PD-1 [24], SHP-1 [25], and IL-11 [26, 27]have been found to have diverse and critical roles in immunity, inflammation, infection, and tumor regression.

In this study, we investigated the expression of RBP7 in hormone receptor-positive breast cancer and analyzed its relationship with clinicopathological features and fatty acid metabolism. We concluded that RBP7 may be a prognostic biomarker and is associated with fatty acid metabolism through the AKT/SREBP1 pathway in hormone receptor-positive breast cancer.

## Materials and methods

### Database analysis

RNA-sequencing expression (level 3) profiles and corresponding clinical information of hormone receptor-positive breast cancer were downloaded from the TCGA dataset (https://portal.gdc.com), including 615 cases of HR + breast cancer and 113 cases of normal tissues. The current-release (V8) GTEx datasets were obtained from the GTEx data portal website (https://www.gtexportal.org/home/datasets), including 459 normal tissues. A total of 2483 immune-related genes were obtained by downloading immune gene data through the ImmPort data portal (http://www.immport.org). A total of 1793 of them were unique elements.

### Screening of independent prognostic differentially expressed genes

On the basis of the RBP7 expression, 615 h + BC samples from TCGA were divided into high (*n* = 308) or low (*n* = 307) groups. The limma package was used to study the differentially expressed genes (DEGs). Differential gene expression between the high and low RBP7 groups was analyzed using the DESeq R package (1.8.3). Heatmaps of differential genes were drawn using the pheatmap R package.

Kaplan-Meier (KM) survival analysis with the log-rank test was used to compare the overall survival (OS) differences between the two groups. The OS-associated genes in HR + BC were screened out by Kaplan-Meier curves using R software version v4.0.3 (The R Foundation for Statistical Computing, 2020). To filter the genes strongly associated with OS, *P* < 0.01 was set before calculating the crossed dataset.

The Venn diagram was drawn by the “VennDiagram package.” Three original datasets of the Venn diagram were DEGs (*n* = 5175), OS-related genes (*P* < 0.01, *n* = 203) and total immune-related genes (*n* = 1793). Univariate and multivariate analyses were performed using the Cox regression method. The forest was drawn by the ‘forestplot’ R package. Genes with *P* < 0.05 were considered potential independent prognostic factors. The log-rank test was used to compare differences in survival between the high and low RBP7 groups. The median survival time was calculated by R software. TimeROC (v 0.4) analysis was used to compare the predictive accuracy of RBP7 mRNA.

### Functional enrichment analysis

The ClusterProfiler package (version: 3.18.0) in R was employed to analyze the GO function of potential targets and enrich the KEGG pathway. The R software ggplot2 package was used to draw boxplots. To further explore signaling pathway enrichment, gene set enrichment analysis (GSEA) was performed between the low and high RBP7 groups using GSEA Java software (https://www.gsea-msigdb.org/gsea/index.jsp). For GSEA, hallmark and C7 gene sets were utilized in this study.

### Construction of a prognostic model

The clinicopathological data matching the HR + BC samples were extracted from the TCGA data resource. Statistical analysis and ggplot2 (v3.3.2) were performed using the R program, and a P value < 0.05 was indicated statistical significance.

The forest map was drawn through the “forestplot” R package to display the P value, HR and 95% confidence interval (CI) of each variable on the basis of RBP7 expression, age and T/N/M stage. The nomogram was built according to the results of multivariate Cox proportional hazards analysis, predicting the overall recurrence rate at 1, 3, and 5 years. The nomogram provided a graphical representation of the independent factors, and the recurrence risk of a single patient can be calculated through the points related to each risk factor in the “rms” R package.

### Tissue samples

To detect the expression of RBP7, a total of 10 tumor samples and 10 normal samples from HR + BC patients who had not undergone therapy before surgical resection were obtained from the First Affiliated Hospital of Soochow University. Consent for participation in the research was duly obtained. Tissue microarrays (TMAs) used for immunohistochemical (IHC) staining analysis were composed of 151 samples, including 28 normal breast tissues, 69 h + tumor tissues and 54 tumor tissues of other molecular types (Bioaitech, Xi’an, China). The Ethics Committee of the First Affiliated Hospital of Soochow University authorized and supervised this study.

### Cell culture

ZR-75-1, ZR-75-30, and BT474 cells were obtained from Abiowell Biotechnology Co., Ltd. (Changsha, China) and cultured in Roswell Park Memorial Institute (RPMI)-1640 medium (#61870-010, Gibco, Rockville, MD, USA) supplemented with 10% fetal bovine serum (#10270-106, Gibco, Rockville, MD, USA). T47D and MCF7 cell lines were obtained from the American Type Culture Collection (ATCC, USA). The T47D cell line was cultured in DMEM/high glucose medium (#SH30022.01B, HyClone, USA), and the MCF7 cell line was cultured in MEM (#SH30265.01B, HyClone, USA). Each medium was supplemented with 10% fetal bovine serum (#10270-106, Gibco, Rockville, MD, USA) and 1% penicillin‒streptomycin (#SH30010, HyClone, USA). Cells were cultured at 37 °C containing 5% CO_2_.

### Construction of stable and transient cell lines

For downregulation of RBP7, siRNAs and shRNA targeting RBP7 were constructed by GenePharma (Suzhou, China). Three potential siRNAs were provided, including RBP7-Homo38 (5′-GCCGACCUCAGCGGUACUUTT − 3′), RBP7-Homo187 (5′- CCACACGAACAGCAGCCUATT − 3′), and RBP7-Homo309 (5′- GGCUCACCUGUAUCCAGAATT − 3′). Lipo8000™ Transfection Reagent (#C0533, Beyotime, Nanjing, China) was used for transient transfection. For RBP7 stable knockdown, MCF7 cells were transfected with shRNA-RBP7 (LV2N(U6/Puro)-RBP7-Homo-309). For upregulation of RBP7, overexpression lentiviruses for RBP7 (pLV3-CMV-RBP7) and control lentiviruses (pCDH-EnCMV-MCS-3×FLA) were purchased from MiaoLingBio (Wuhan, China). Polybrene (#H9268, Sigma, USA) was used for transfection. Follow-up experiments were carried out.

### Quantitative reverse transcriptase polymerase chain reaction (qRT‒PCR)

The RNA-Quick Purification Kit (#RN001, Yishan Biotechnology, Shanghai, China) and PrimeScriptTM RT Master Mix Kit (#RR036A; Takara, Dalian, China) were used for total RNA extraction and cDNA synthesis, respectively. An AceQ qPCR SYBR Green Master Mix (without ROX) kit (#Q121-02-AA; Vazyme, Nanjing, China) was used to perform real-time quantitative PCR. The specific scheme was conducted in accordance with the instructions of the reagent manufacturer. The relative gene expression levels were normalized to GAPDH and calculated by the 2^−∆∆Ct^ method. The sequences of primers used for qRT‒PCR are as follows:

GAPDH: Forward, 5’-AATCCCATCACCATCTTCCA-3’

GAPDH: Reverse, 5’-TGGACTCCACGACGTACTCA-3’

RBP7: Forward, 5’-CTCAGCGGTACTTGGACCC-3’

RBP7: Reverse, 5’-CGAGTGGCAAAGTCAATACCT-3’

### Cell counting Kit-8 (CCK-8) assay

Cell viability was determined by Cell Counting Kit-8 (CCK-8) (#C6005, NCM Biotech, Suzhou, China) assay. A total of 2000 breast cancer cells were seeded in 96-well plates and incubated for 24 h, 48 h, 72 and 96 h. After that, 10 µL CCK-8/well was added to the culture medium and incubated for another 2 h. The OD450 was determined on an enzyme plate analyzer.

### Western blotting

Tumor cells were lysed with RIPA lysis buffer (#P0013B, Beyotime, Shanghai, China) supplemented with protease and phosphatase inhibitor cocktail (#P1045, Beyotime, Shanghai, China) and then sonicated. After centrifuging the lysate, the supernatant was collected and the concentration was determined with the bicinchoninic acid method (BCA Protein Assay Kit, #P0012S, Beyotime, Shanghai, China). Total protein (20 µg) was separated by 12.5% SDS/PAGE gel (#P2013, NCM, Suzhou, China) and then transferred to polyvinylidene fluoride (PVDF) membranes (#10,600,023, GE, USA), blocked with 5% skim milk (#P0216, Beyotime, Shanghai, China), and incubated with primary antibodies, such as mAbs anti-RBP7 Ig (1:1000, #14541-1-AP, Proteintech, Wuhan, China), anti-GAPDH Ig (1:50000, #60004-1-Ig, Proteintech, Wuhan, China), anti-AKT Ig (1:1000, #4691; Cell Signaling Technology, Shanghai, China), anti-p-AKT Ig (1:2000, #4060; Cell Signaling Technology, Shanghai, China) and anti-SREBP1 Ig (1:1000, #14088-1-AP, Proteintech, Wuhan, China), overnight at 4 °C. The membrane was then incubated with the secondary antibody (1:5000, #SA00001-1, #SA00001-2, Proteintech, Wuhan, China) at room temperature for 1 h, and ECL Western blot reagent (#36222ES, YEASEN, Shanghai, China) was added after washing. A Chemi DocTM MP Imaging System was used for detection.

### Transwell assays

24-well Transwell plates (8 μm pore size) were used for invasion and migration assays. For migration assays, a total of 5 × 10^4^ cells resuspended in serum-free medium were seeded in the inserts, while 800 µL of medium containing 10% FBS was added into the lower chamber.

In the invasion assay, Matrigel (#C0371, Beyotime, Shanghai, China) was added to the Transwell chamber 2 h in advance, and then the cell suspension was added. The remaining steps were the same as above.After 24–48 h, invaded cells were fixed with 4% paraformaldehyde (#P0099, Beyotime, Shanghai, China) and stained with 1% crystal violet (#C0121, Beyotime, Shanghai, China). Six random fields per well were photographed by microscopy and counted.

### Colony formation

After transfection, 1000 cells were seeded in 6-well plates and incubated in a cell incubator at 37 °C with 5% CO_2_ and saturated humidity for 14 days. Cells were fixed using 4% paraformaldehyde and stained with 1% crystal violet for colony enumeration.

### Wound healing assays

To generate a confluent monolayer, 5 × 10^5^ cells were seeded in six-well plates and cultured. Wound areas were scraped using 200-µl pipette tips, followed by three washes with PBS to eliminate debris. Subsequently, culture media without FBS was added. The closure of the wounds was observed and captured using an inverted microscope at 0 and 48 h.

### Cell cycle analysis

To analyze the cell cycle, the collected cells were fixed with ethanol at -20 °C for 24 h. After two washes with 1×PBS, the cells were incubated in the dark at room temperature with PI/RNase staining solution (#C1052, Beyotime, Shanghai, China) for 15 min. The DNA content of each cell phase was determined using a flow cytometer.

### Xenograft mouse models

Animal experiments were carried out in accordance with the guidelines and approval of the Institutional Animal Care and Use Committee of Soochow University (Suzhou, China). For the experiment, five-week-old SPF NCG female mice (Gempharmatech Co., Ltd, Nanjing, China) were randomly allocated into three groups: kd-RBP7 group, WT (wild-type) group and oe-RBP7 group, with 5 mice in each group. Subcutaneous injections of kd-RBP7, wild-type and oe-RBP7 MCF7 cells were administered to the mice on the same side. After 36 days, all mice were humanely sacrificed through cervical dislocation, and the tumors were collected for further measurement.

### Nile red staining and oil red O staining

Oil red O staining was used for lipid droplet quantification. For Oil Red O staining, frozen cells were fixed with 4% paraformaldehyde for 10 min and then incubated with Oil Red O solution (six parts Oil Red O stock solution and four Parts H_2_O; Oil Red O stock solution was 0.5% Oil Red O in 100% isopropanol) (#C0157S, Beyotime, Shanghai, China) for 15 min. Nile red is a fluorescent stain for intracellular lipid droplets. For Nile red staining, the fixed cells were incubated with 0.1 µg/mL Nile red (#7385-67-3, Angene, Nanjing, China) for 15 min. DAPI (#C1005, Beyotime, Shanghai, China) was used to stain the nucleus.

### Free fatty acid measurement

The free fatty acid determination was carried out using a free fatty acid content assay kit (#BL869B, Biosharp, Hefei, China). The extraction solution was added according to the number of cells or the quality of the tumor samples according to the instructions. The cells were lysed with an ultrasound instrument and centrifuged to obtain the supernatant. A testing group and a control group were established according to the instructions of the reagent kit. After sufficient shaking, the supernatant was taken, and the absorbance was measured at 715 nm using an enzyme-linked immunosorbent assay. The absorbance value was substituted into the standard curve equation to obtain the free fatty acid content.

### Statistical analysis

Each experiment in the present work was performed in triplicate. The statistical analysis was conducted using SPSS software (version 13.0). Student’s t test was used to compare the two groups, and ANOVA was used to compare multiple groups. A significance level of *P* < 0.05 was used to determine statistical significance.

## Results

### RBP7 was expressed at low levels in HR + BC and related to OS in patients with HR + BC

**Fig. 1:**
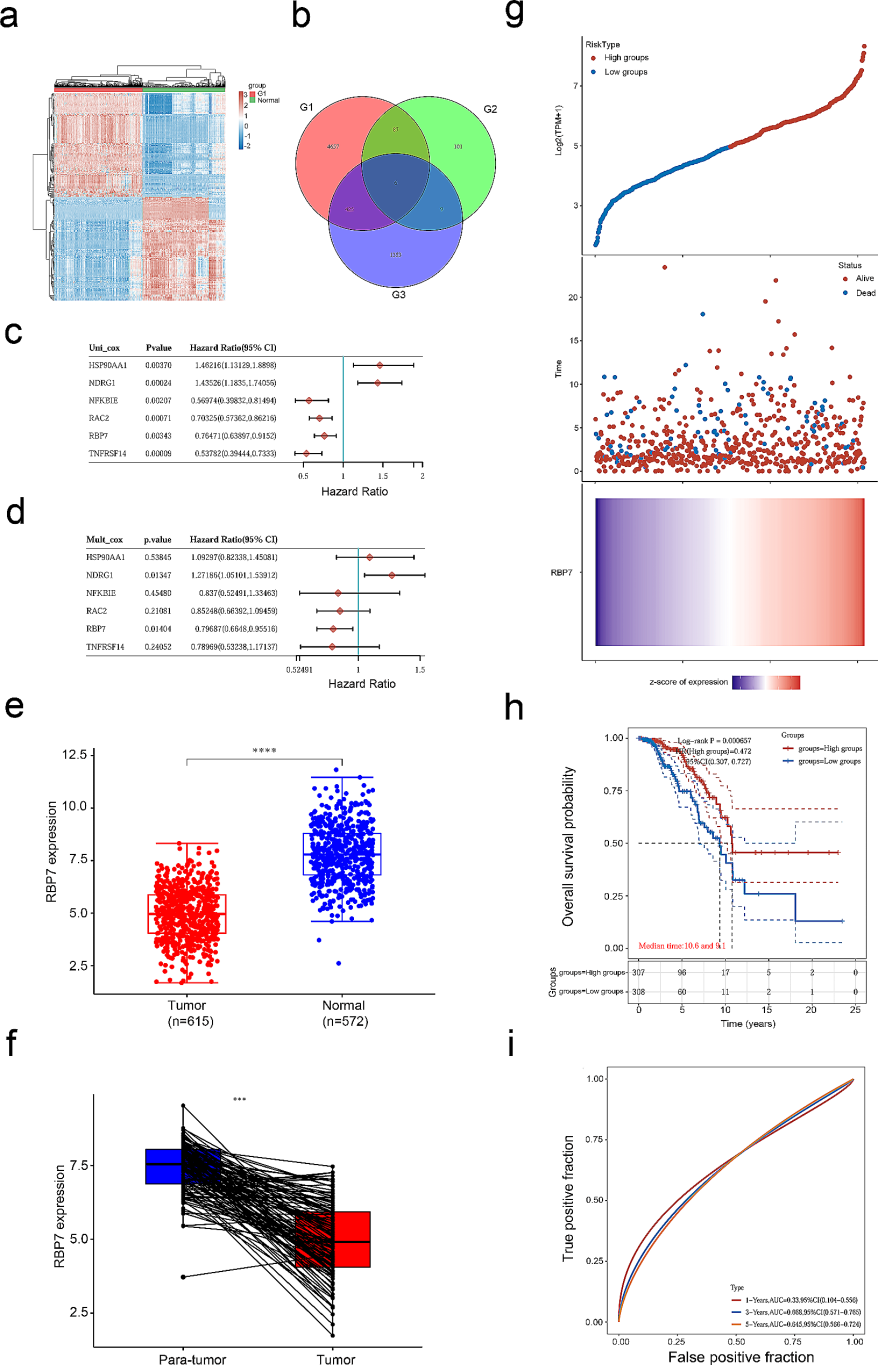
Expression and prognosis value of RBP7 in HR + BC. **a** Heatmap of the differentially expressed genes (DEGs) between HR + BC (G1) and normal tissues. Red: significantly up-regulated DEGs; blue: significantly down-regulated DEGs. **b** Venn diagram of 6 differentially expressed genes were found in the intersection of three databases. G1: DEGs between HR + BC and normal tissues from TCGA-BRCA dataset (*n* = 5175). G2: OS-associated genes (*P*<0.01, *n* = 203). G3: Immune-related genes screened from ImmPort data portal (*n* = 1793). **c** Univariate Cox regression analysis of the 6 DEGs. **d** Multivariate Cox regression analysis of the 6 DEGs. **e** RBP7 mRNA expression levels in TCGA HR + BC tumor tissues and TCGA and GTE_X_ normal tissues. **f** RBP7 mRNA expression levels in HR + BC tumor tissues and para-tumor tissues in TCGA. **g** Distribution of the HR + BC patients based on risk scores and survival time and status of the HR + BC patients. Red: high risk groups; blue: low risk groups. **h** Kaplan-Meier survival curves of high- and low-RBP7 patients in HR + BC cohorts. **i** ROC curves with calculated AUCs for OS prediction in HR + BC.

The different expression levels of hormone receptor-positive breast cancer and normal control samples from TCGA were compared. Through differential expression analysis, 5175 differentially expressed genes were obtained, 3728 of which were highly expressed, while 1447 differentially expressed genes were downregulated. The heatmap shows the top 50 upregulated genes and 50 downregulated genes (Fig. 1a).

To investigate the survival-associated and immune-related genes of DEGs in hormone receptor-positive breast cancer, 6 genes (HSP90AA1, NDRG1, NFKBIE, RAC2, RBP7, TNFRSF14) were screened using a Venn map (Fig. 1b). Univariate and multivariate analyses showed that NDRG1 and RBP7 were independent prognostic factors for OS, and RBP7 might act as a tumor suppressor gene in hormone receptor-positive breast cancer (Fig. 1c, d).

A total of 615 patients from TCGA were divided into two groups based on the expression of RBP7 (high or low). The samples from TCGA (615 h + BC and 113 normal tissues) and GTEx (459 normal tissues) were analyzed, and the results revealed that RBP7 expression was downregulated in hormone receptor-positive BC tissues compared with that in normal tissue samples (Fig. 1e). Similar results were obtained in hormone receptor-positive breast cancer tissues and paired adjacent normal tissues (Fig. 1f).

According to the median risk score, the patients were divided into a low-risk group and a high-risk group. Through the log rank test, the Kaplan‒Meier survival curve suggested that patients in the high-risk group had better OS than those in the low-risk group (*P*<0.001, HR = 0.472, 95% CI 0.307–0.727) (Fig. 1g). The median survival times of the high and low RBP7 expression groups were 10.6 years and 9.1 years, respectively (Fig. 1h). The ROC curve showed that the AUCs of 1-, 3-, and 5-year survival in patients with hormone receptor-positive BC were 0.33, 0.668 and 0.645, respectively (Fig. 1i).

### Enrichment analyses of RBP7 in HR + BC

**Fig. 2:**
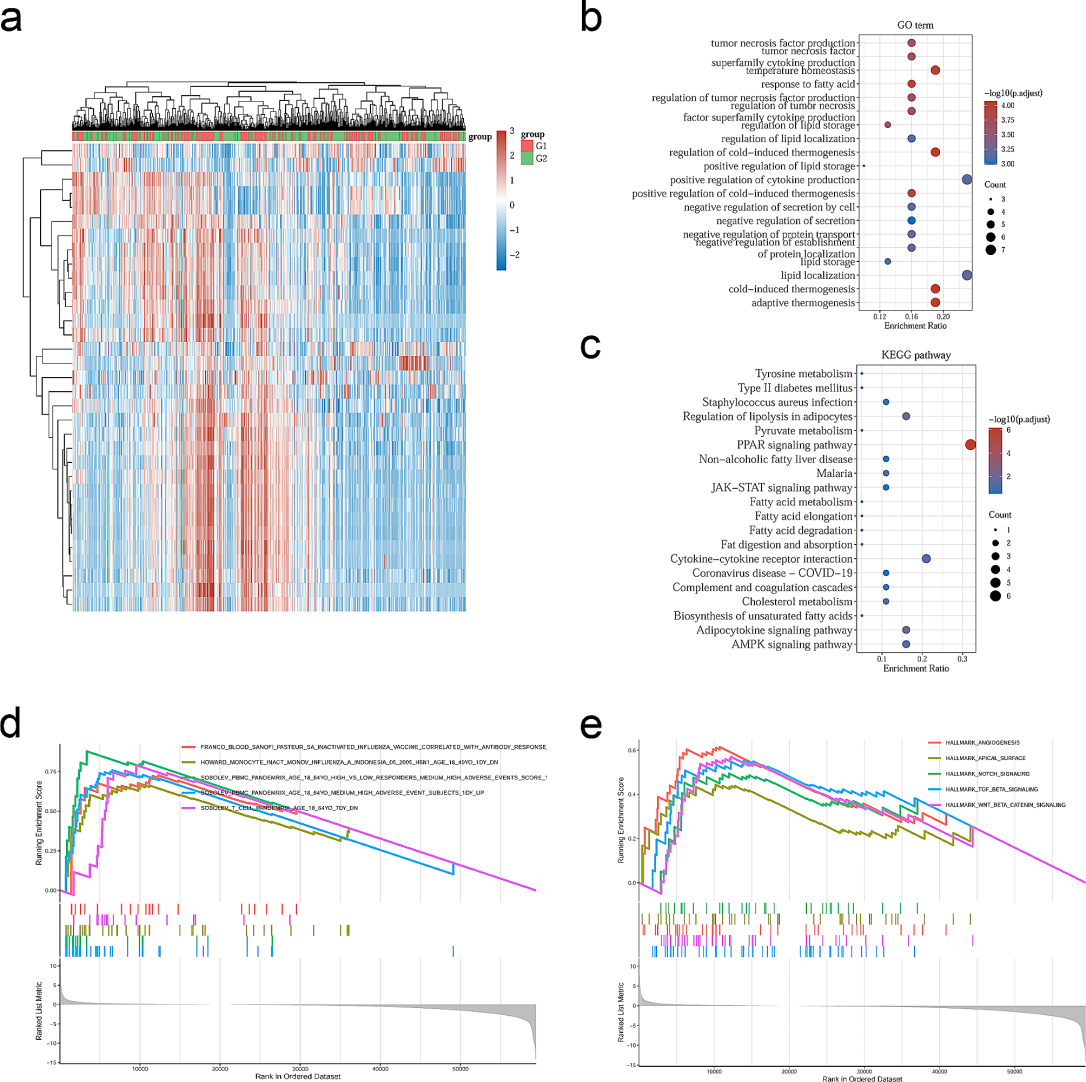
Potential functions of RBP7. **a** Heatmap of the DEGs between HR + BC with high RBP7 and low RBP7 expression. **b** Dotplot of top 20 enriched GO terms of molecular function, cellular component and biological process. **c** Dotplot of top 20 enriched KEGG pathways. **d,e** Gene set enrichment analysis (GSEA) of the top 5 enriched pathways in RBP7-high expression phenotype in the C7 and HALLMARK collection from TCGA datasets

The heatmap shows the differential gene expression between the high and low RBP7 groups (Fig. 2a). For a better understanding of the molecular underpinnings of the common DEGs, these DEGs were subjected to GO and KEGG enrichment analysis.

Functional enrichment analysis illustrated that these genes were actively associated with functions of response to fatty acids, factor superfamily cytokine production, and positive regulation of cytokine production in GO terms (Fig. 2b), and the genes associated with fatty acid degradation and the PPAR signaling pathway in KEGG terms are shown in Fig. 2c. For the C7 collection, a rich set of immune function genes was observed in the group with upregulated RBP7 expression (Fig. 2d). The HALLMARK data illustrated that some signaling cascades consisting of TGF-β signaling and WNT-β signaling were abundant in the group with high expression of RBP7 (Fig. 2e).

### RBP7 protein was down-regulated in HR + BC tissues and cell lines

**Fig. 3:**
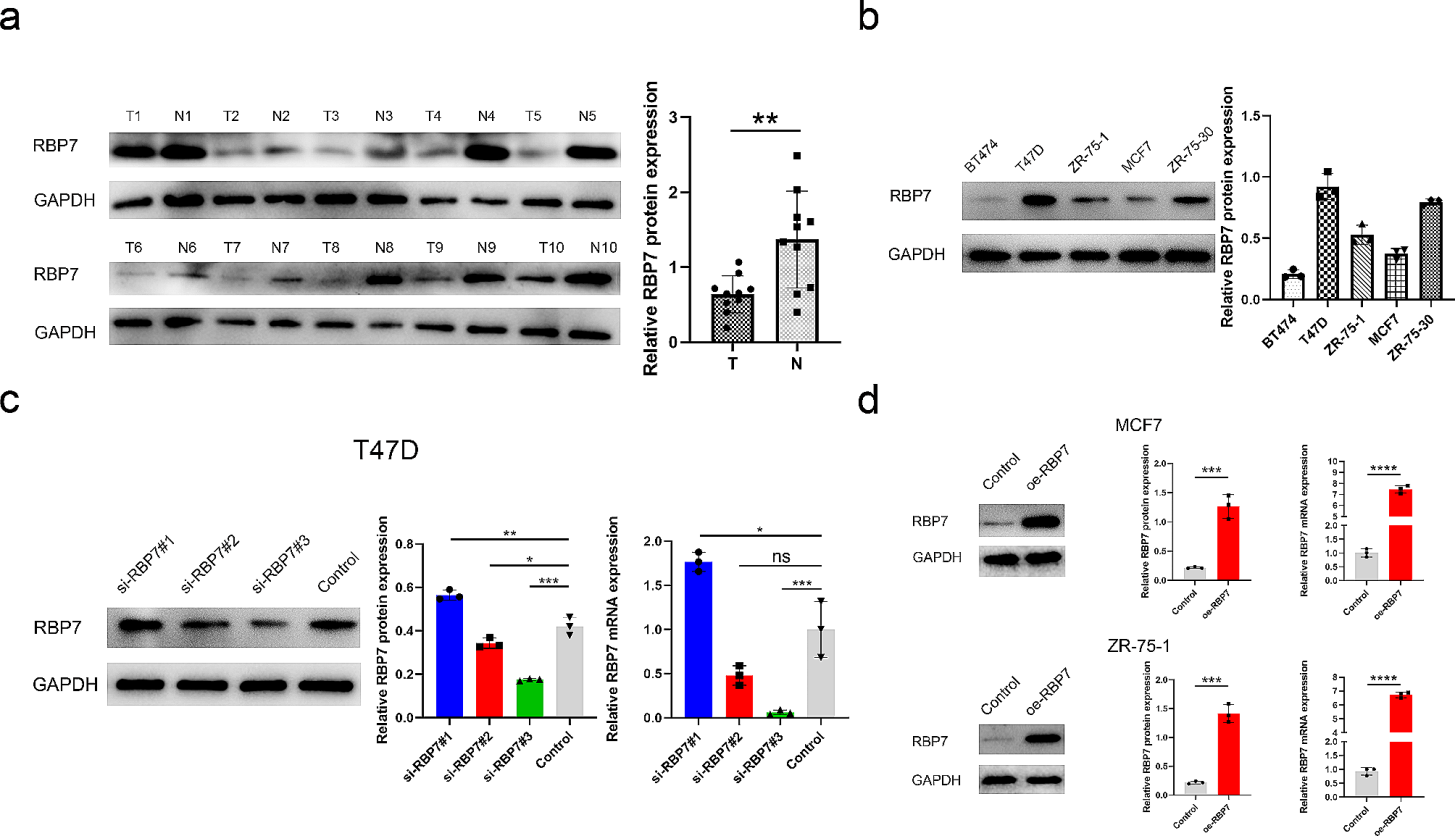
RBP7 expression in HR + BC clinical samples and cell lines. **a** Western blotting of the RBP7 level in HR + BC clinical samples, T: tumor, N: para-tumor. **b** Western blotting of the RBP7 level within 5 h + BC cells. **c** After downregulation of RBP7 expression in the T47D cell lines, the RBP7 expression was detected by Western blot and qRT-PCR. **d** After upregulation of RBP7 expression in the MCF7 and ZR-75-1 cell lines, the RBP7 expression was detected by Western blot and qRT-PCR. Data were represented as means ± S.D. of at least three independent experiments. *P < 0.05, **P < 0.01, ***P < 0.001

To determine the expression level of RBP7, western blotting was performed in 10 paired HR + BC tissues and adjacent normal tissues. The expression of RBP7 protein in tumors was significantly lower than that in adjacent normal tissues (Fig. 3a). Levels of RBP7 protein expression were evaluated in HR + BC cell lines. Most HR + BC cell lines showed obviously low expression of RBP7, while the T47D cell line showed high expression of RBP7 (Fig. 3b). The T47D cell line was selected to downregulate RBP7 expression. Western blotting and qPCR were used to verify the efficiency of knockdown. (Fig. 3c). MCF7 and ZR-75-1 cell lines were selected to induce RBP7 overexpression (Fig. 3d).

### RBP7 suppressed cell proliferation

**Fig. 4:**
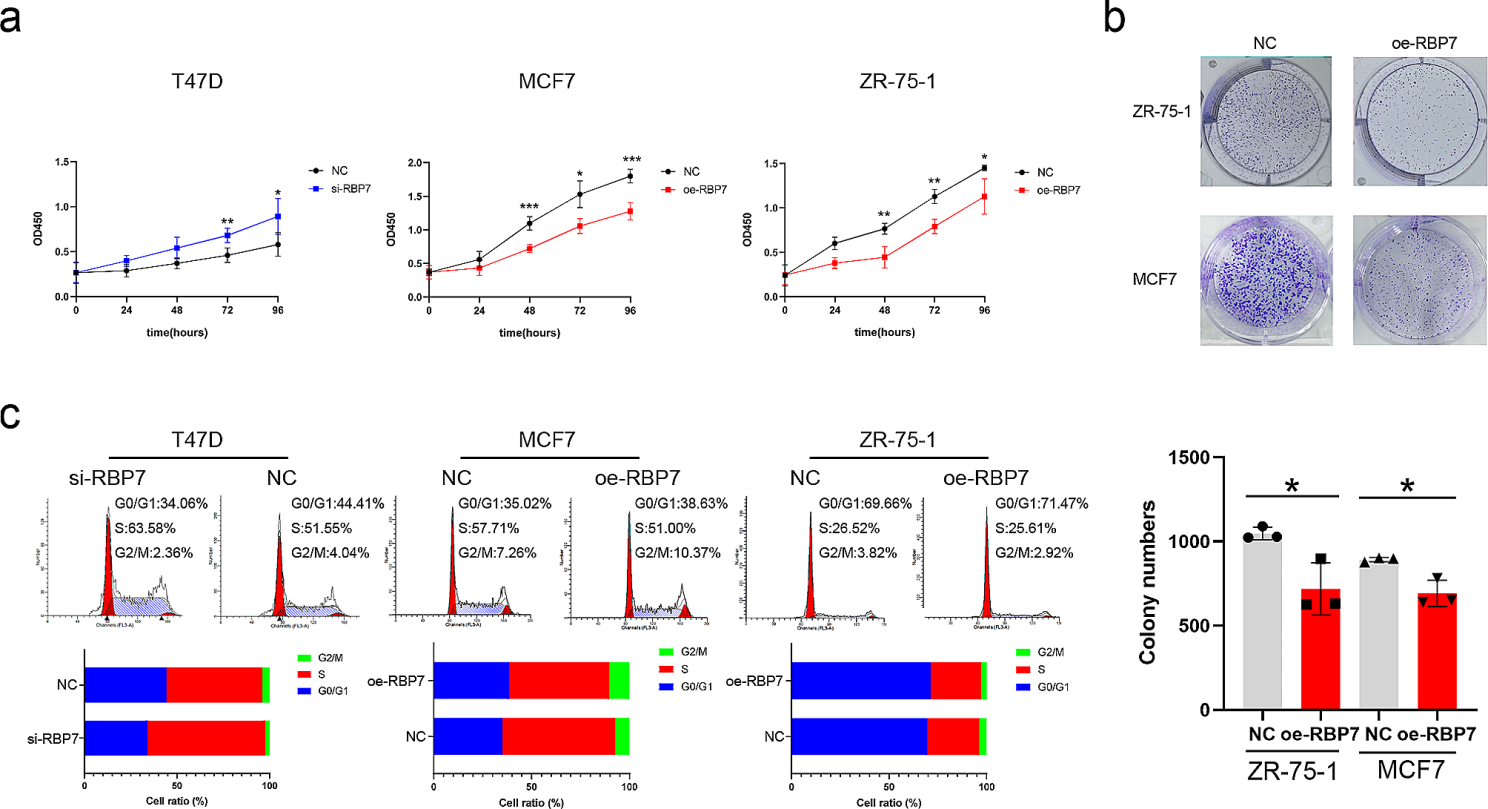
RBP7 inhibited the proliferation of HR + BC cells. **a** CCK8 assay was applied to determine the proliferation of HR + BC cells with different expression of RBP7. **b** Effects of RBP7 overexpression on the colony formation of HR + BC cells. **c** Effects of RBP7 difference on cell cycle distribution. Data were represented as means ± S.D. of at least three independent experiments. *P < 0.05, **P < 0.01, ***P < 0.001

The CCK-8 results suggested that the downregulation of RBP7 promoted cell proliferation in T47D cells. The upregulation of RBP7 suppressed cell proliferation in MCF7 and ZR-75-1 cells (Fig. 4a). In addition, up-regulated RBP7 expression markedly reduced colony growth and colony formation ability within the 2D plates of MCF7 and ZR-75-1 cells (Fig. 4b). Cell cycle assays demonstrated that compared to cells in the NC groups, MCF7 and ZR-75-1 cells in the oe-RBP7 groups had an increased proportion of cells in the G0/G1 phase. Correspondingly, it was observed that in the si-RBP7 T47D cell line, the ratio of cells in G0/G1 phase was decreased (Fig. 4c).

### RBP7 inhibited HR + BC cell migration and invasion

**Fig. 5:**
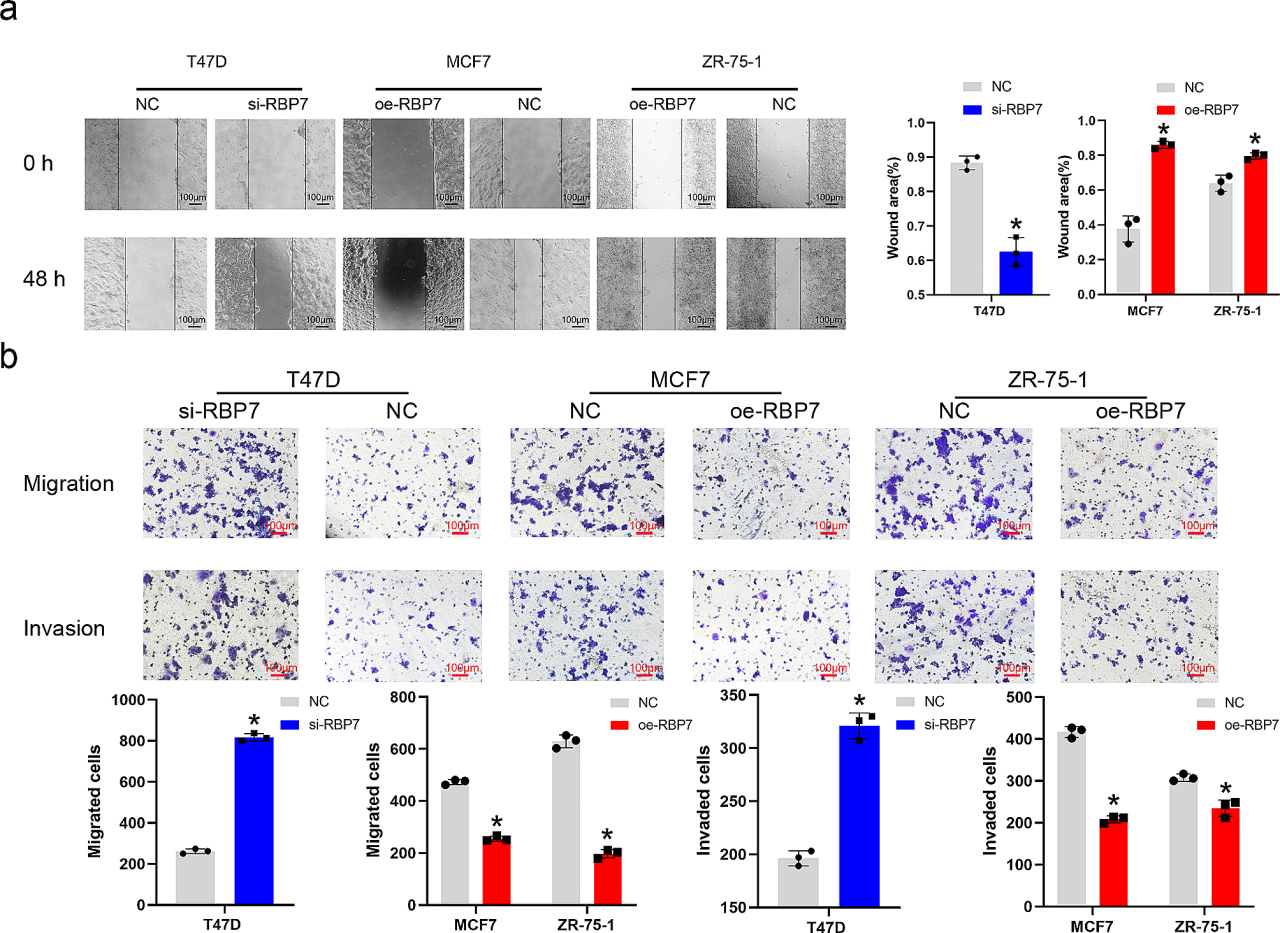
RBP7 inhibited the migration and invasion of HR + BC cells. **a** Effects of RBP7 difference on migration of HR + BC cells in wound-healing assays. **b** Effects of RBP7 difference on migration and invasion of HR + BC cells in transwell assays. Data were represented as means ± S.D. of at least three independent experiments. *P < 0.05, **P < 0.01, ***P < 0.001

To explore how RBP7 affected HR + BC cell invasion and migration, wound healing assays and Transwell assays were carried out. According to wound healing assays, overexpression of RBP7 in MCF7 and ZR-75-1 cell lines significantly inhibited cell migration compared with the control group. Down-regulated expression of RBP7 induced T47D cell migration compared with that in the control group (Fig. 5a). Transwell experiments showed that upregulation of RBP7 reduced the number of migrated and invaded cells, while downregulation of RBP7 could reverse the effect (Fig. 5b).

### RBP7 reduced fatty acid content in HR + BC cells by inhibiting the AKT/SREBP1 pathway

**Fig. 6:**
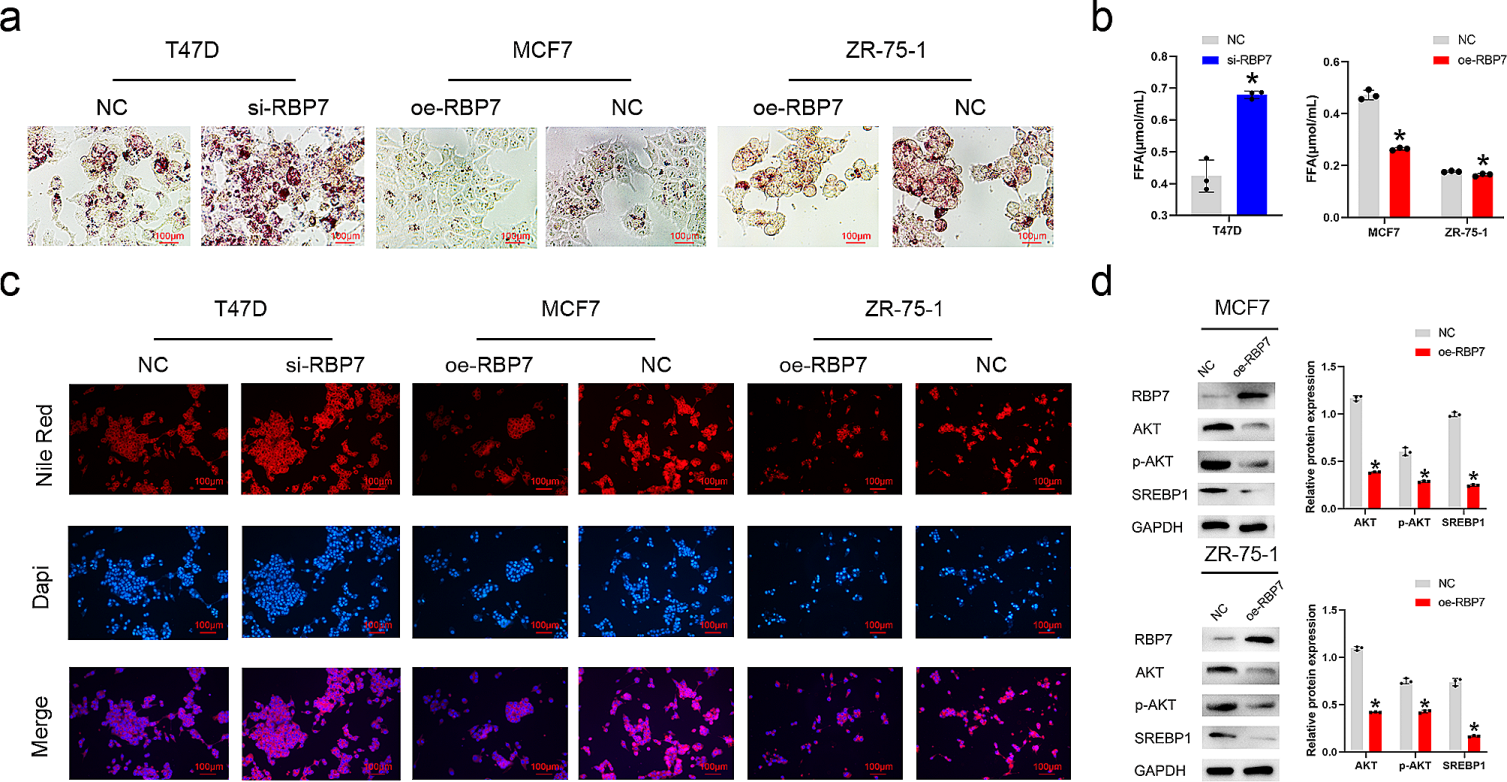
RBP7 reduced the fatty acid content in HR + BC cells and inhibited AKT/SREBP1 pathway. **a** Effects of RBP7 difference on neutral fat content tested by Oil Red O staining. **b** Effects of RBP7 difference on FFA using FFA content assay kit. **c** Effects of RBP7 difference on cellular lipid tested by Nile Red staining. **d** Effects of RBP7 difference on the expression of AKT, p-AKT and SREBP1 protein. Data were represented as means ± S.D. of at least three independent experiments. *P < 0.05, **P < 0.01, ***P < 0.001

By staining the cells with oil red O, we observed a significant decrease in lipid accumulation in the cells overexpressing RBP7 compared with the control. Meanwhile, lipid accumulation was up-regulated in the si-RBP7 T47D cell line compared with the NC group (Fig. 6a). Consistently, Nile red staining indicated that downregulation of RBP7 led to increased cellular lipid accumulation and that overexpression of RBP7 led to cellular lipid reduction (Fig. 6b). Quantitative testing of fatty acids showed that decreased expression of RBP7 resulted in an increase in intracellular free fatty acid content, while overexpression of RBP7 resulted in decreased FFA (Fig. 6c). Western blot analysis revealed that AKT, p-AKT, and SREBP1 were markedly decreased by overexpressing RBP7 in HR + BC cells, demonstrating that RBP7 reduced fatty acid content by inhibiting the AKT/SREBP1 pathway (Fig. 6d).

### RBP7 inhibited HR + BC xenografted Tumor Growth in NCG mice

**Fig. 7:**
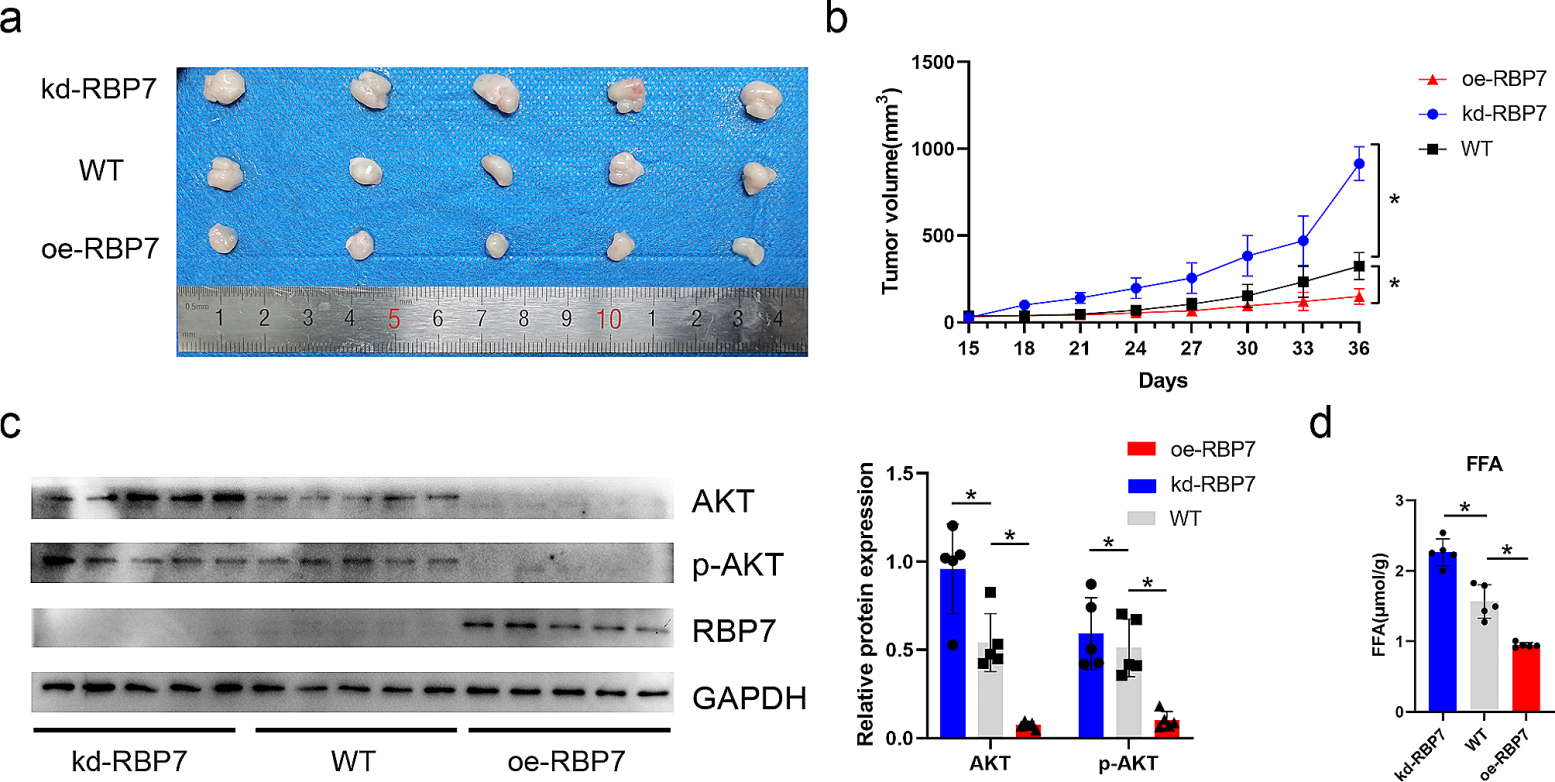
RBP7 inhibited HR + BC xenografted tumor growth and reduced tumor FFA content. **a** Image of representative resected tumors from xenograft NCG mice on the 36th day. **b** Growth curves of kd-RBP7, oe-RBP7 and WT MCF7 groups. **c** Effects of RBP7 difference on the expression of AKT and p-AKT protein in xenografted tumor. **d** Effects of RBP7 difference on FFA in xenografted tumor. Data were represented as means ± S.D. of at least three independent experiments. *P < 0.05, **P < 0.01, ***P < 0.001

To investigate the effect of RBP7 in vivo, WT MCF7 cells, kd-RBP7 MCF7 cells and oe-RBP7 MCF7 cells were subjected to subcutaneous inoculation into NCG mice. Overexpression of RBP7 significantly decreased xenograft tumor volume, while downregulation of RBP7 could reverse the effect (Fig. 7a, b). RBP7 inhibited AKT and p-AKT in xenografted Tumors (Fig. 7c). Quantitative testing of free fatty acids showed that RBP7 led to a decrease in FFA (Fig. 7d). These findings suggested that RBP7 suppressed HR + BC tumor growth in vivo and reduced fatty acid content.

### The expression of RBP7 was negatively correlated with T-stage and Ki67 score in HR + BC

**Fig. 8:**
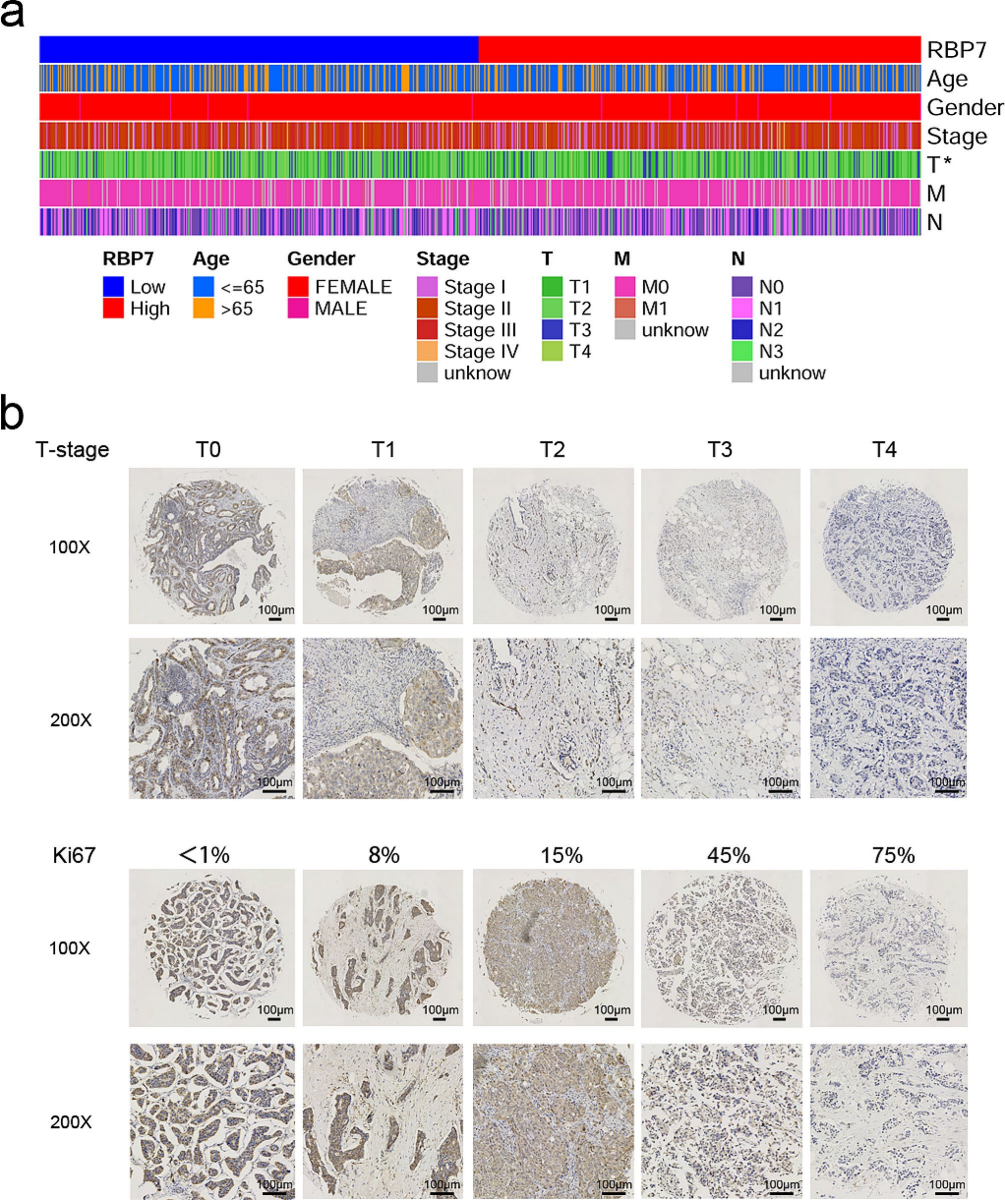
RBP7 expression was related to T-stage and Ki67 score. **a** The association between clinicopathological features and RBP7 mRNA expression levels in HR + BC patients from TCGA. ( *P < 0.05) **b** Typical images of RBP7 immunohistochemical staining of HR + BC tissues with different T-stages and Ki67 scores from the tissue microarray

From the TCGA database, the relationship between RBP7 expression and age, stage, and TNM stage was displayed by a heatmap. The results indicated that patients with high RBP7 expression had lower tumor T stage (Fig. 8a). From tissue microarray analysis, the relationship between RBP7 expression and age, stage, T stage, and N stage is displayed in Table 1. The results showed that patients with high RBP7 expression had lower tumor T-stage. In addition, RBP7 expression was negatively correlated with Ki67 score (Fig. 8b). The expression of RBP7 in normal breast tissues was higher than that in HR + breast cancer tissues (Table 2).

**Table 1:** Table 1 Correlation between RBP7 expression and clinicopathological features in HR + BC patients

Title	Group	RBP7 expression(%)		Total	χ2	*P*
		high	low			
Stage	I	1(2.63)	0(0.00)	1(1.45)	1.981	0.852
	IIA	9(23.68)	5(16.13)	14(20.29)		
	IIB	7(18.42)	6(19.35)	13(18.84)		
	IIIA	18(47.37)	16(51.61)	34(49.28)		
	IIIB	1(2.63)	2(6.45)	3(4.35)		
	IIIC	2(5.26)	2(6.45)	4(5.80)		
Ki-67	low	20(52.63)	25(80.65)	45(65.22)	5.906	0.015*
	high	18(47.37)	6(19.35)	24(34.78)		
T	I-II	35(92.11)	23(74.19)	58(84.06)	4.087	0.043*
	III-IV	3(7.89)	8(25.81)	11(15.94)		
N	0	11(28.95)	5(16.13)	16(23.19)	1.575	0.209
	I-III	27(71.05)	26(83.87)	53(76.81)		
Age	≥ 60	7(18.42)	5(16.13)	12(17.39)	0.062	0.803
	<60	31(81.58)	26(83.87)	57(82.61)		
Total		38	31	69		

* *P* < 0.05 ** *P* < 0.01

**Table 2:** Table 2 Comparison of RBP7 expression in HR + BC and normal breast tissues

Title	Group	RBP7(%)		Total	χ2	*P*
		high	low			
Tissue	normal	26(46.43)	2(4.88)	28(28.87)	19.902	0.000**
	tumor	30(53.57)	39(95.12)	69(71.13)		
Total		56	41	97		

* *P* < 0.05 ** *P* < 0.01

### Construction of a nomogram

**Fig. 9:**
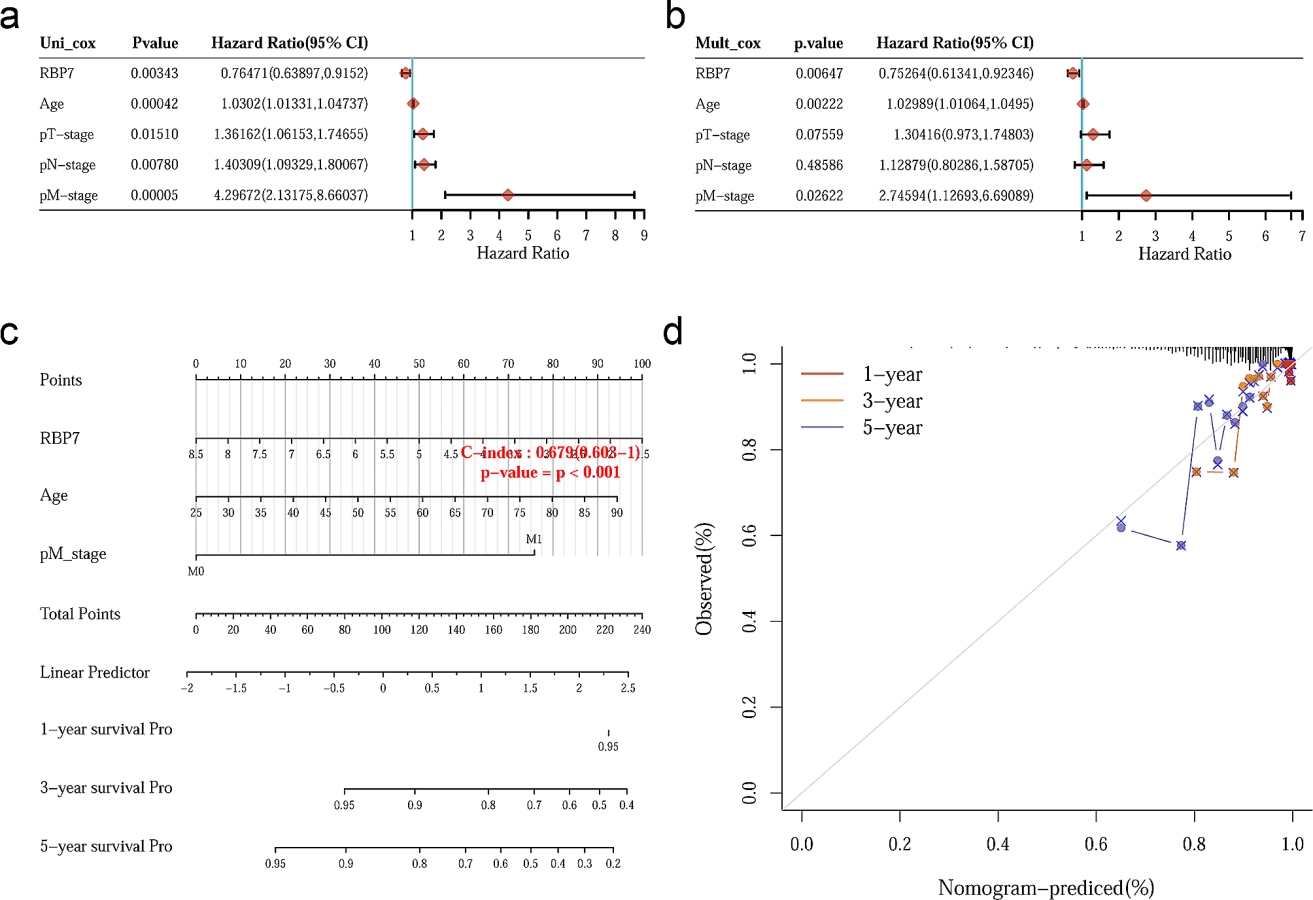
Visualization of the prognostic model. **a-b** Prognostic factors for OS were analyzed using univariate and multivariate analysis according to Cox regression model. **c** A nomogram based on RBP7 expression, M stage and age for predicting overall survival. **d** The correction plot of 1-year, 3-year and 5-year OS predicted by the nomogram

For further exploration of the comprehensive value of the risk model combined with other factors, univariate Cox regression analysis was performed to determine the clinical characteristics related to prognosis. The data were from the TCGA database. The results showed that age (*P* < 0.001), T stage (*P* = 0.0151), N stage (*P* = 0.0078), M stage (*P* < 0.001) and RBP7 expression (*P* = 0.0034) were significantly associated with prognosis (Fig. 9a). Then, multivariate Cox analysis was applied to analyze the independent prognostic factors. The results suggested that age (*P* = 0.0022), M-stage (*P* = 0.0262) and RBP7 expression (*P* = 0.0065) were independent prognostic factors in predicting overall survival (Fig. 9b). To better predict the prognosis of patients with hormone receptor-positive BC in different years after diagnosis, we constructed a new nomogram based on clinical features and RBP7 expression. In the nomogram, the higher the total points, the worse the OS (Fig. 9c). The calibration curve showed the accuracy of the nomogram (Fig. 9d).

## Discussion

According to the investigation, breast cancer ranks first in accounting for approximately 30% of all new tumors in women [28]. By the end of 2020, a total of 7.8 million living women had been diagnosed with breast cancer in the past five years, making it the most common cancer in the world [29].Since 2005, HR + breast cancer has increased by approximately 0.9% per year in white women, 1.7% per year in Hispanic women, 2.3% per year in Asian/Pacific (API) women, and 2.5% per year in American Indian/Alaskan native (AIAN) women [30].

The prognosis of HR + breast cancer patients was previously thought to be the best among all subtypes of breast cancer; however, with the development of anti-HER2 targeted therapy for HER2 + breast cancer and intensive therapy for triple-negative breast cancer, the prognostic advantage of HR + early breast cancer patients is no longer obvious. About 40% of HR + early breast cancer patients treated with tamoxifen (TAM) will recur, and the risk of distant metastatic recurrence ranges from 10 to 41% depending on tumor size and lymph node metastatic status [31, 32]. However, unlike other subtypes of breast cancer, more than 50% of HR + early breast cancer patients’ recurrence occurs 5 years after diagnosis, and this risk of recurrence can extend beyond 10 years [33]; even stage IA patients with a better prognosis have a high risk of long-term recurrence, with a 20-year cumulative distant recurrence rate of 13% [32]. Therefore, the discovery of effective prognostic factors and therapeutic targets is important for therapeutic development in HR + breast cancer.

Over the years, generous studies have identified numerous tumor suppressor genes that play a crucial role in regulating cell growth and preventing the progression of cancer [34]. Tumor suppressor genes can serve as biomarkers for cancer diagnosis [35] and prognosis [36] and as targets for therapies [34]. Recent studies have explored the value of RBP7 in anticancer [20] and tamoxifen resistance [19] in HR + BC. However, the mechanism of RBP7 in BC remains unknown.

In this study, we analyzed the TCGA and GTEx databases and found that normal tissues or para-tumor tissues tended to have relatively high levels of RBP7 expression compared with HR + BC tissues. Higher RBP7 was associated with better OS of patients, indicating that RBP7 is a tumor suppressor gene in HR + BC. We collected 10 groups of HR + BC tissues and paired normal tissues to verify the expression of RBP7 in HR + BC tissues. The results demonstrated that RBP7 was significantly downregulated in HR + BC tissues compared to normal tissues. Similar findings were observed in tissue microarrays, suggesting a significant decrease in RBP7 expression in HR + BC patients. In addition, combined with the clinicopathological features of HR + BC patients from TCGA and tissue microarrays, RBP7 expression was negatively correlated with the T stage and Ki67 score of HR + BC. The HR + BC patients with higher RBP7 expression had a lower T stage and a lower Ki67 score, demonstrating that RBP7 might be a novel prognostic factor for patients with HR + BC.

Next, in vivo and in vitro experiments were performed to explore the roles of RBP7 in regulating the proliferation, migration and invasion of HR + BC cells. We found that overexpression of RBP7 could significantly reduce the proliferation, invasion, and migration of HR + BC cells. Moreover, increased expression of RBP7 led to a higher percentage of cells in the G0/G1 phase. Conversely, the knockdown of RBP7 enhanced cell proliferation, migration and invasion while reducing the proportion of cells in the G0/G1 phase. The xenograft tumor mouse models suggested that overexpression of RBP7 resulted in a substantial decrease in tumor size. These findings provide evidence that RBP7 may have an antitumor impact on HR + BC cells, making it a potential targeted therapeutic molecule for patients with HR + BC.

Breast cancer has a unique tumor microenvironment compared to other solid tumors, surrounded by numerous adipocytes capable of generating and releasing fatty acids and adipokines [37]. Fatty acid metabolism plays an important role in hormone positive breast cancer. Fatty acid metabolism is related to estrogen synthesis and signal transduction [38]. The development of hormone positive breast cancer is closely related to the role of estrogen, and fatty acid metabolism can affect the synthesis and signal transduction of estrogen, thus regulating the development of breast cancer [39]. Fatty acid metabolism is related to the proliferation and metastasis of breast cancer cells [40, 41]. Fatty acid metabolism can provide energy and nutrients for breast cancer cell growth and proliferation, and promote the growth and metastasis of breast cancer [42, 43]. Fatty acid metabolism is also associated with drug resistance in treatment. Some studies have shown that abnormal fatty acid metabolism is related to the resistance of breast cancer to hormone therapy [44, 45]. Therefore, the sensitivity of hormone positive breast cancer to treatment can be improved by regulating fatty acid metabolism. From enrichment analysis of RBP7 in HR + BC in KEGG and GO terms, it was observed that there were significant pathways enriched in relation to fat function and lipid metabolism. These pathways included the regulation of lipid localization, lipid storage, fatty acid metabolism, fatty acid degradation, and the biosynthesis of unsaturated fatty acids. Therefore, we detected the neutral fat content through staining in HR + BC cells after knocking down or overexpressing RBP7. The results demonstrated that lipid accumulation was significantly enhanced after knocking down RBP7. Overexpression of RBP7 showed reverse impacts, indicating that RBP7 may affect the biological characteristics of HR + BC by affecting fat metabolism. Fatty acids are important molecules involved in energy metabolism, membrane synthesis, and signaling pathways [46]. The impact of RBP7 on fatty acid levels was quantitatively measured in HR + BC cells and xenografted tumors. The findings revealed that RBP7 effectively decreased the FFA content in HR + BC. The results above suggested that RBP7 may play an antitumor role by enhancing fatty acid metabolism in HR + BC. RBP7 might be a novel therapeutic target to enhance the prognosis of HR + BC patients, decrease drug resistance, and prevent recurrence.

The AKT pathway plays a significant role in breast cancer development and progression [47]. Hyperactivation of the AKT pathway promotes cell survival, proliferation, and resistance to apoptosis, contributing to tumor growth, metastasis, and treatment resistance in breast cancer [48]. AKT signaling can modulate the expression and activity of transcription factors involved in the regulation of fatty acid metabolism [49]. Targeting the AKT pathway and fatty acid metabolism has emerged as a potential therapeutic strategy for cancer treatment. The expression of AKT in HR + BC cells and xenografted tumors was examined through WB analysis, and we found a significant decrease in AKT and p-AKT expression in MCF7 and ZR-75-1 cells overexpressing RBP7, indicating that RBP7 could inhibit the AKT signaling pathway in HR + BC cells.

**Fig. 10:**
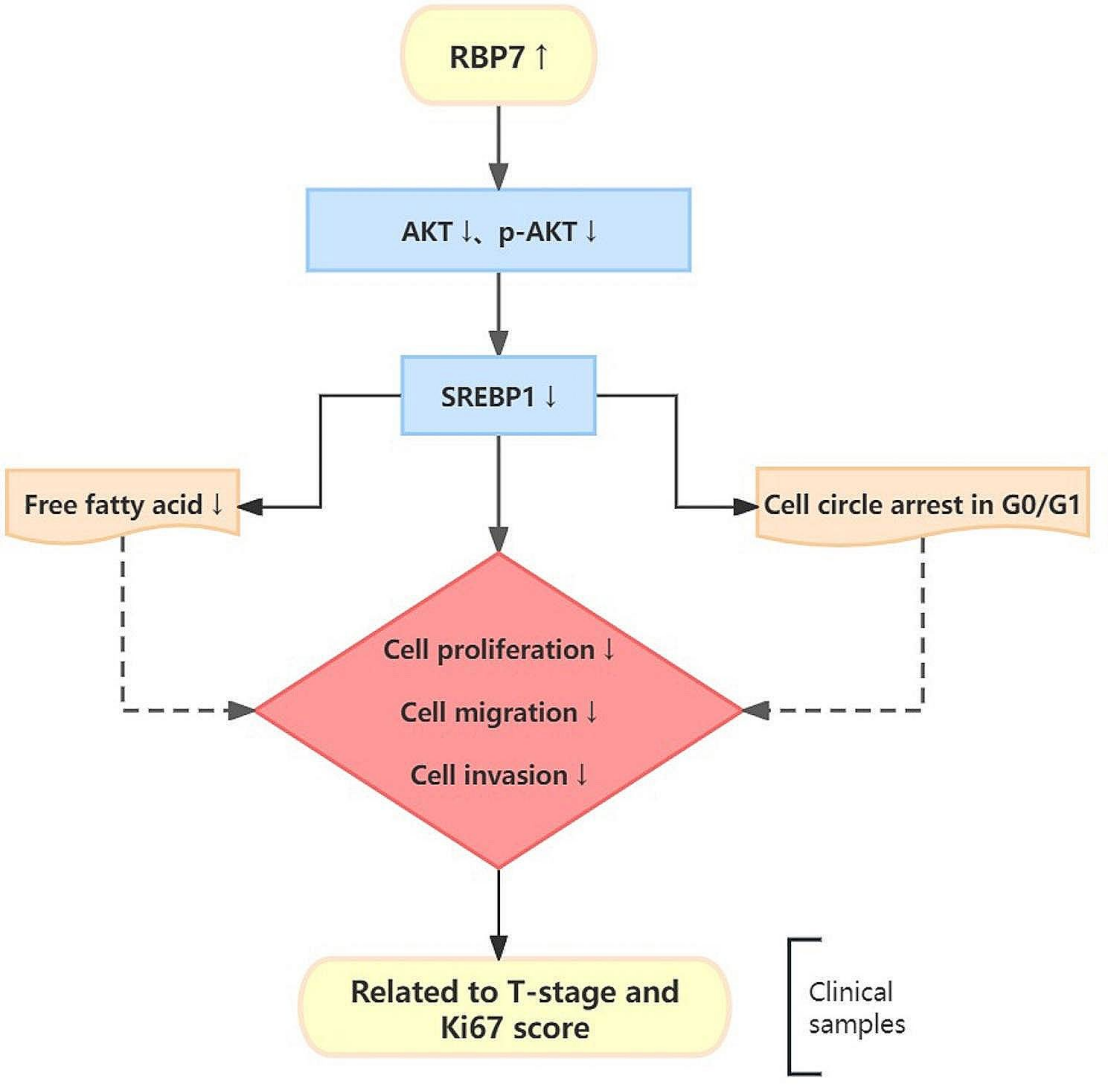
Schematic representation of proposed mechanism of action of RBP7 induced G0/G1 phase arrest and FFA reduction as well as inhibition of proliferation, migration and invasion in HR + BC cells. Upregulation of RBP7 decreased the levels of AKT, p-AKT and SREBP1, leading to FFA content reduction and G0/G1 phase arrest. Overexpression of RBP7 could suppress cell proliferation,migration and invasion. Furthermore, the correlation between RBP7 expression and tumor T-stage and Ki67 score has been validated through clinical sample validation

SREBP1 (Sterol regulatory element-binding protein 1) is a transcription factor that plays a crucial role in the regulation of lipid metabolism [50]. The dysregulation of SREBP1 in cancer cells promotes increased lipid synthesis, which is essential for the rapid growth and proliferation of cancer cells [51]. Furthermore, SREBP1 has been found to interact with other oncogenic signaling pathways, such as the PI3K/Akt/mTOR pathway, to promote cancer cell growth and survival [52]. In this study, we found that the expression of SREBP1 in HR + BC cells overexpressing RBP7 was also significantly reduced compared to that in the control group. The above results revealed that RBP7 could inhibit the AKT/SREBP1 signaling pathway, thereby promoting the decline in fatty acid content and inhibiting HR + BC progression. According to above results we herein proposed a model for potential mechanism of action of RBP7 in HR + BC as shown in Fig. 10.

## Conclusion

In summary, we identified RBP7 as an anti-oncogene in HR + BC in this study. Reduced RBP7 expression was associated with tumor progression and poor prognosis. Mechanistically, RBP7 inhibited the AKT/SREBP1 pathway and reduced the fatty acid content, thus regulating the proliferation, cell cycle, migration and invasion of HR + BC cells. The results obtained in this study suggest that RBP7 could be used as a potential prognostic biomarker and therapeutic target for HR + BC patients.

## Data Availability

All data generated or analyzed during this study are included in this published article.

## Abbreviations

BC: Breast cancer
HR: Hormone receptor
BPs: Cellular binding proteins
CRBP: Cellular retinol binding proteins
PPARγ: Peroxisome proliferator-activated receptorγ
IRGs: Immune system-related genes
DEGs: Differentially expressed genes
KM: Kaplan-Meier
OS: Overall survival
GSEA: Gene set enrichment analysis
GO: Gene Ontology
KEGG: Kyoto Encyclopedia of Genes and Genomes
TCGA: The Cancer Genome Atlas
CI: Confidence interval
TMAs: Tissue microarrays
IHC: Immunohistochemical
DMEM: Dulbecco’s modifed Eagle’s medium
MEM: Modifed Eagle’s medium
RPMI: Roswell Park Memorial Institute
qRT-PCR: Quantitative reverse transcriptase polymerase chain reaction
CCK-8: Cell Counting Kit-8
ANOVA: Analysis of variance
cDNA: Complementary DNA
SDS/PAGE: Sodium dodecyl sulfate polyacrylamide gel electrophoresis
BCA: Bicinchoninic acid method
PVDF: Polyvinylidene fluoride
PI: Propidium iodide
FFA: Free fatty acid
OE: Overexpression
KD: Knock down
SREBP1: Sterol regulatory element-binding protein 1
AUC: Area Under the Curve
API: Asian/Pacific Islander
AIAN: American Indian/Alaskan native
HER2: Human epidermal growth factor receptor 2
